# How to Strengthen Patients’ Meaning Response by an Ethical Informed Consent in Psychotherapy

**DOI:** 10.3389/fpsyg.2019.01747

**Published:** 2019-07-31

**Authors:** Manuel Trachsel, Martin grosse Holtforth

**Affiliations:** ^1^Institute of Biomedical Ethics and History of Medicine, University of Zurich, Zurich, Switzerland; ^2^Department of Psychology, University of Bern, Bern, Switzerland; ^3^Division of Psychosomatic Medicine, University Hospital Bern, Bern, Switzerland

**Keywords:** psychotherapy, ethics, meaning response, informed consent, common factors, treatment rationale

## Abstract

Healthcare professionals including psychotherapists are legally and ethically obliged to ensure informed consent for the provided treatments comprising type and duration or potential benefits and possible risks (e.g., side effects) among others. In the present contribution, we argue that as potential benefit, informed consent can foster the patient’s meaning response. Moerman’s notion of the meaning response as the physiological or psychological effects of meaning in the course and treatment of an illness is a useful concept in explaining the effects of communicating a treatment rationale as part of the informed consent procedure. The more compelling the rational explanation of the targeted treatment effects including an explanatory model and a model of unique and common change mechanisms, the stronger the meaning response is expected to be resulting in increased hope and positive expectations with regard to the treatment.

## Introduction: The Moral Obligation of Informed Consent in Psychotherapy

*Informed consent* provides the major *legal and moral legitimation* for physical medicine as well as psychological interventions including psychotherapy independent of the setting, be it inpatient or outpatient, individual, couples, or group therapy (American Psychiatric Association, 1998; Beauchamp and Childress, 2013; Trachsel et al., 2015a,b). For that reason, informed consent is a necessary prerequisite for any intervention and a moral duty, reflecting the individual’s right to self-determination (moral principles: respect for autonomy; dignity), patient well-being, and protection (moral principle: beneficence), as well as doing no harm (moral principle: non-maleficence). By meeting all those moral values, informed consent has been named a central element of *patient-centered care* in a contribution in the JAMA (Krumholz, 2010). Patient-centered care has been named as one of six core attributes of a high-quality health care system by the Institute of Medicine Committee on Quality of Health Care in America is defined as “providing care that is respectful of and responsive to individual patient preferences, needs, and values, and ensuring that patient values guide all clinical decisions” (IOM, 2001, p. 40).

Accordingly, informed consent is a central tenet of the American Medical Association (2006), which holds that “the physician has an ethical obligation to help the patient make choices from among the therapeutic alternatives consistent with good medical practice” (Opinion 8.08) and that “withholding medical information from patients without their knowledge or consent is ethically unacceptable” (Opinion 8.082).

According to Beauchamp and Childress (2013), informed consent must include the following components: patient *decision-making capacity* (DMC) and *voluntariness*; *disclosure of relevant information* by the healthcare professional; and the *statement of consent* itself. With regard to DMC, the following criteria are widely accepted (Grisso and Appelbaum, 1998): (1) ability to understand the relevant information; (2) ability to appreciate the disorder and the consequences of the situation; (3) ability to reason about treatment choices; and (4) ability to communicate a choice. These criteria have been criticized for their exclusive focus on cognition while neglecting emotional processes (for a summary of this debate, see Hermann et al., 2016). As DMC often changes with symptom fluctuation over time and across different situations (Trachsel et al., 2015a,b), its validity can generally be assumed only for the time of assessment.

For decades, it has been argued that informed consent should become a standard element of psychiatric and psychotherapeutic practice, as in other fields of medicine (Beahrs and Gutheil, 2001). The American Psychiatric Association (1998), p. 24, declared that a “psychiatrist shall not withhold information that the patient needs or reasonably could use to make informed treatment decisions […].” Similarly, the Meta Code of Ethics of the European Federation of Psychologists’ Associations (2005) requires “clarification and continued discussion of the professional actions, procedures and probable consequences of the psychologist’s actions to ensure that a client provides informed consent before and during psychological intervention” (Art. 3.1.3).

Informed consent should include among others type, duration, and costs of a treatment, or potential benefits and possible risks such as side effects. As it is one of the most important insights in medicine since the time of Hippokrates that medical treatments can harm, side effects are well known and studied for many treatments, and should therefore be communicated to patients as part of the informed consent process. In psychotherapy, however, side effects have been less studied although it has been always obvious that mere speaking can have negative effects (e.g., Linden and Schermuly-Haupt, 2014). Despite this fact, there is no consensus what psychotherapy side effects are and how to assess them (see Linden et al., 2018). Furthermore, side effects are often entangled with symptoms of the disorder itself or unsuccessful therapy (Hoffmann et al., 2008). However, these are no good reasons to get around informing patients that side effects also occur in psychotherapy, even if the therapist does her or his best and acts according to evidence-based principles of good psychotherapy.

Furthermore, it should also be disclosed if there exist multiple relationships, which have the potential to lead into conflicts of interest. Multiple relationships occur when a therapist is in a professional role with a client while simultaneously being in another role with the same person, a person closely associated with or related to the client, or promises to enter into a secondary relationship with any of those mentioned persons in the future (American Psychological Association, 2016). For example, in countries in which outpatient psychotherapy is not covered by statutory health insurances, this can lead to financial interests of the therapist with regard to the psychotherapy fee, which should be disclosed in the process of informed consent.

At its best, the most important contents of the informed consent should be put to record and the patient should be provided with it in written form. However, this is not mandatory in most jurisdictions and oral informed consent is legally sufficient.

Regrettably, informed consent for psychotherapy remains non-routine (Trachsel et al., 2015a,b); instead, Dsubanko-Obermayr and Baumann (2010) suggested that agreement in relation to treatment and its elements is most often tacitly assumed but rarely explicated.

Beyond legal and moral duty, informed consent can benefit the psychotherapeutic process and outcome, especially through the healthcare professional’s disclosure of relevant information. In line with the apparent consensus that certain *common factors* are relevant to outcome (see e.g. Wampold and Imel, 2015), and based on the principle that informed consent requires adequate disclosure of treatment information, it can be argued that the relevance of common factors for psychotherapy’s effects should also be conveyed to patients (see also Gaab et al., 2016). If patient autonomy is to be respected, therapists should provide their patients with the following information. (1) Agreement about the goals and tasks of therapy may support treatment success. (2) A good working alliance between patient and therapist can foster good psychotherapy processes and outcomes, and patients should feel supported, encouraged, and understood by their therapist. (3) Patients should be aware that their view of the therapist and the proposed treatment may also help or hinder therapy (Blease et al., 2016a,b). For example, agreement about goals and tasks is important in augmenting mutual trust and is central to the therapeutic alliance as a key component of a successful outcome (Lambert and Barley, 2002; Horvath et al., 2011).

As part of the disclosure of relevant information in the context of informed consent, psychotherapists are also required to provide a plausible *treatment rationale*. This may include both an explanatory model (why a certain person is having a certain problem) and a model of treatment (what should be done to solve this person’s problem)—that is, assumed and empirically supported unique and common mechanisms of change. With some patients, however, it might take considerable time and more than one or two sessions to develop an appropriate explanatory model as part of an informed consent. Comorbidity with several mental disorders (including personality disorders) in concert with multiple somatic disorders may further complicate and prolong the assessment period before a meaningful case formulation may be possible. In these cases, the development of an explanatory model and preparation of an individual case formulation may become a treatment goal in its own right and should be discussed as a part of the informed consent process (see also Tryon, 2019).

In the present contribution, we argue that as potential benefit, informed consent can foster the patient’s meaning response. Moerman’s notion of the meaning response as the physiological or psychological effects of meaning in the course and treatment of an illness is a useful concept in explaining the effects of communicating a treatment rationale as part of the informed consent procedure. The more compelling the rational explanation of the targeted treatment effects including an explanatory model and a model of unique and common change mechanisms, the stronger the meaning response is expected to be resulting in increased hope and positive expectations with regard to the treatment.

In the following sections, we will present Moerman’s notion of the meaning response, its relation to the treatment rationale, its contribution as a way to realize common factors in psychotherapy, as well as its relation to therapy outcome. Earlier, we present our conclusion in the last section of the present article, we provide a fictional case example illustrating how the meaning response may contribute to the realization of common psychotherapy factors.

## The Meaning Response

Provision of a plausible treatment rationale, including assumed and empirically supported unique and common mechanisms of change, meets the legal and moral demands of informed consent. On the other hand, a plausible treatment rationale is all the more important because “[c]linical experience and an accumulating body of research suggests that clients who enthusiastically buy into a cognitive-behavioral treatment rationale show more favorable outcomes” (Addis and Carpenter, 2000, p. 147). It seems likely that this effect also plays a role in other forms of psychotherapy.

But how might a convincing treatment rationale foster good psychotherapy outcomes? It is sometimes assumed that providing a rational explanation of treatment effects makes a favorable outcome more likely by the patient’s *meaning response*. Moerman and Jonas defined meaning response as “the physiologic or psychological effects of meaning in the origins or treatment of illness” (Moerman and Jonas, 2002, p. 472), elaborating this as “the idea of ‘meaning’ to which people, when they are sick, often respond” (Moerman and Jonas, 2002, p. 471). Moerman contended that “the most important aspect of any medical experience is its *content* […] [b]ut the content of medicines is not the only thing that counts” (Moerman and Jonas, 2002, p. 47) and that “The *form* of medical treatment, not just its *content*, can have a dramatic effect on human wellbeing” (Moerman and Jonas, 2002, p. 66).

Meaning making is central to every treatment, including psychotherapy. Moerman (2002), p. 94, stated that “[t]o me, it sounds […] reasonable to say that psychotherapy evokes meaning responses”. This is a thought that Frank (1993) already had in mind when he wrote his seminal book *Persuasion and Healing* in 1961, in which he “proposed the then-radical notion that all forms of psychotherapy, as different as they were on the surface, worked because they contained similar elements” (Moerman, 2002, p. 93)—for example, a helping relationship involving the therapist’s thoughtful listening. According to Moerman, Frank said in one interview that “[p]sychotherapy relies on the fact that human beings react not to the facts or events themselves *but to the meanings of the facts as they interpret them.* Psychotherapy is the transformation of the meanings that patients attribute to events from negative to positive” (Moerman, 2002, p. 96). In similar vein, Irving Kirsch observed: “The point is that meaning is the essence of psychotherapy. It is through meaning that treatment effects are supposed to be brought about” (Kirsch, 2010, p. 164). Kirsch referenced Albert Ellis’s rational emotive therapy—the first cognitive therapy for emotional problems—which fundamentally presupposed “that the way we feel does not depend on the events that happen to us, but rather on the meaning these events have for us” (Kirsch, 2010, p. 164). Kirsch went on: “[w]hat we need is a way to activate a therapeutic meaning response in clinical practice, and to do so without deceiving people or playing tricks on them […]” (Kirsch, 2010, p. 165).

Meaning making seems central to verbalizing negative experiences, whether in oral or written form. In many studies, James W. Pennebaker found that writing or talking about traumatic events consistently helped patients to experience positive health effects of some sort (see for example, Pennebaker, 1997). Moerman (2002) drew on later studies by Pennebaker that showed why some “trauma writers” experienced better outcomes: “[…] they write better stories—stories that are more coherent, more persuasive, better organized. In a word, their stories seem (to me) to be more *meaningful*” (Moerman, 2002, p. 98).

Moerman and Jonas (2002) provided several examples from pharmacology to illustrate the notion of meaning response. For example, referring to Branthwaite and Cooper (1981), they stated: “Branded aspirin worked better than unbranded aspirin, which worked better than branded placebo, which worked better than unbranded placebo. […] Aspirin relieves headaches, but so does the knowledge that the pills you are taking are ‘good’ ones” (Moerman and Jonas, 2002, p. 471). In their view, it seems reasonable to characterize these effects (other than those of the branded aspirin) as meaning responses, as the study participants seemed to assign meaning to the intervention. In addition, Moerman and Jonas noted the common and widely replicated “meanings” that most people share: “(1) Red means ‘up,’ ‘hot,’ ‘danger,’ while blue means ‘down,’ ‘cool,’ ‘quiet’ and (2) two means more than one” (Moerman and Jonas, 2002, p. 472).

In sum, Moerman’s (2002) introduction of the concept of meaning response serves to replace the term “placebo effect.” Indeed, Brody (1980) had previously characterized Adler and Hammett’s (1973) meaning model as replacing the concept of placebo: “In this model, the subjective sense of *meaning* in the illness experience is factored into (1) *system formation,* or the providing of a coherent explanation of the illness consistent with the patient’s world view, and (2) *group formation*, or the gathering of a supportive, caring group around the patient” (Brody, 1980, p. 115). While Brody outlined the semantic and interpersonal components of meaning, Moerman mainly focused on the semantic component.

If, as potential benefit, informed consent fosters the patient’s meaning response this could facilitate an active engagement of patients in a recommended treatment which, from a motivational perspective, can be understood in the context of the transtheoretical stages of change model as a step from the stages of precontemplation, contemplation or preparation to the stage of action (see e.g., Prochaska and Norcross, 2018).

Despite the plausibility of the effects of meaning making cited above, neither Moerman (2002) nor Moerman and Jonas (2002) clarified their use of the term *meaning* or the concept of meaning response. According to Baumeister (1991), meaning can be broadly defined as a “mental representation of possible relationships among things, events, and relationships. Thus, meaning connects things” (Baumeister, 1991, p. 15). On that basis, it is suggested here that if one takes meaning as the network of mental representations associated with a process of meaning making does not necessarily happen at a conscious level; as Park observed, “Meaning making has been conceptualized as both automatic and unconscious processes” (Park, 2010, p. 259), so that meaning need not necessarily be conceptualized solely within the realm of consciously held beliefs.

Returning to the conscious aspect of meaning in the context of psychotherapy, a rational explanation can potentially bestow meaning on a certain treatment by enriching the patient’s semantic network regarding the concept of psychotherapy and related concepts and association; the more compelling the explanation of treatment effects for the individual, the more intense the meaning response is likely to be (Moerman and Jonas, 2002). For example, it is known that, among medical interventions, patients find surgery especially meaningful. This may be because surgeons enjoy one of the highest credibility ratings among physicians, and because surgical procedures can mostly be explained in accessible rational terms—for instance, “We will reconnect your upper arm bone with two screws, and then you just have to wear a cast for three weeks” (for example, see Kaptchuk et al., 2000). In contrast, explanations of pharmaceutical effects commonly engender weaker meaning responses because they are more complex and less palpable—for example, “The drug will inhibit the inflammation process by reducing the production of inflammatory substances in your body” (Moerman and Jonas, 2002).

We contend that in psychotherapy, the mechanisms of change are often even more abstract and less immediately accessible than many pharmacological terms or other medical concepts. Fortunately, these mechanisms are to a large extent common to many psychotherapeutic interventions, making it all the more important to embed accessible explanations of common factors within a convincing treatment rationale in order to engender the best possible meaning response.

The next section explicates how a patient’s meaning response can be supported by providing a convincing treatment rationale during the process of informed consent to psychotherapeutic treatment, and how this can help to realize common factors associated with a favorable psychotherapeutic outcome.

## Realization of Common Factors in Psychotherapy Through Meaning Response

Common factors can be defined as “those elements of psychotherapy that are so frequently present in different psychotherapeutic treatments that they cannot be considered to be restricted to one school of psychotherapy” (McAleavey and Castonguay, 2015, p. 295). In an empirical study based on expert ratings, Tracey (2003) identified three distinct clusters of common factors: bond, information, and structure, respectively referring to the therapeutic relationship, the provision of specific information and conceptual knowledge, and the explicit or implicit organization of psychotherapy as an interpersonal encounter. For example, if a therapist provides detailed information and a rational explanation of the proposed therapy as part of the informed consent procedure, this may enhance the patient’s perception of the therapist as a competent and trustworthy specialist, fostering positive expectations of process and outcome (see for example, Frank, 1968; Bloch, 2006) that relate mostly to positive treatment outcomes (Lambert, 2013). It follows that a patient’s meaning response to the provision of information at the beginning of psychotherapy is central to the realization of common factors. The patient’s meaning response can therefore be seen as an important precondition for a favorable psychotherapeutic process and outcome.

To optimize the meaning response, it is important that the informed consent process, including provision of a treatment rationale, is customized for the individual patient. According to Adler and Hammett (1973), provision of a treatment rationale is “system formation, or the providing of a coherent explanation of the illness consistent with the patient’s world view” (Brody, 1980, p. 115).

Because the mechanisms of change in psychotherapy are considered more complex than in other fields of medicine and the treatment process more individual, provision of a rationale for psychotherapeutic treatment must usually be more customized than, for example, in the case of pharmacological or surgical treatment (Trachsel et al., 2015a,b). However, in so doing, psychotherapists have a moral duty to remain within the range of evidence-based treatments (e.g., Blease et al., 2016a,b). Trachsel et al. (2015a,b) put it as follows:

For example, in a symptom-focused psychotherapy, goals, risks, and procedures are more concretely nameable beforehand, whereas the goals of an insight-oriented psychotherapy need to be more openly formulated. Consequently, due to the less foreseeable course of an insight-oriented therapy, a more complex and contingent [informed consent] may be required at intake, whereas a more straightforward [informed consent] may be pursued for symptom-focused psychotherapy that more closely resembles [informed consents] for pharmacological treatment. (776).

## Ms. Hope’s Meaning Response

The following fictional case example serves to illustrate how the meaning response may contribute to the realization of common psychotherapy factors. On a friend’s recommendation, Ms. Hope entered psychotherapy because of sleeping problems and feelings of depression. She had no prior personal experience of psychotherapy and knew little or nothing about it or what effects to expect. At the beginning of the first session, she said “For me, it is just worth a shot. If I don’t benefit, it will certainly not cause any harm.” However, the therapist knew that a favorable course of psychotherapy depended crucially on positive outcome expectations and conveyed to the patient her conviction that evidence-based psychotherapy was the appropriate treatment. The therapist sought to ensure that initiating psychotherapy would make sense to Ms. Hope in order to motivate her to open up and to work hard on her problems. To foster these positive expectations, the therapist explained in clear and simple language how psychotherapy is thought to work in general—for example, that it is crucial to establish a confidential and trusting psychotherapy relationship to facilitate discussion of difficult topics. In addition, she explained how the specific evidence-based psychotherapy approach she practiced could help Ms. Hope (based on assumed and empirically supported efficacy factors). Additionally, the therapist provided Ms. Hope with a summary of the results of relevant psychotherapy research, showing that evidence-based psychotherapy is likely to be successful for most patients with problems similar to her own. These explanations formed part of the informed consent procedure that the psychotherapist is legally and ethically obliged to provide. As the explanations begin to make sense to Ms. Hope, beginning psychotherapy becomes meaningful for her—in other words, Ms. Hope exhibits a meaning response. Developing trust in the psychotherapist and her approach, the patient gains confidence that the psychotherapy will help her to deal with her sleeping problems and feelings of depression.

## Conclusion

We contend that Moerman’s notion of meaning response as the physiological or psychological effects of meaning in the course and treatment of illness should be taken into account by healthcare professionals, including psychotherapists. To engender a strong meaning response in patients, psychotherapists should provide detailed and customized information in support of an accessible rational explanation of the proposed psychotherapy as part of the informed consent procedure. One factor that may also become a necessary part of the treatment rationale is the explication of treatment expectations (Constantino et al., 2011). For example, if a patient has the “passive” treatment expectation that the therapist will fix his or her problems without a contribution on his or her part, an active engagement in possibly effortful treatment procedures is less likely. To foster positive outcome expectancies, it might be necessary to first teach the patient that his or her active engagement in potentially effortful therapy procedures will be necessary to reach the best possible outcome. In addition, establishing an according task agreement will be an important part of establishing a good therapeutic alliance as a booster of good outcome (Flückiger et al., 2018; Westermann et al., in press).

By strengthening both, the patient’s perception of the psychotherapist as a competent and trustworthy specialist, and the patient’s anticipation that his or her active personal engagement in psychotherapy will be crucial for reaching the best possible outcome, it is also likely to foster the positive process and outcome expectations shown to be associated with positive treatment outcomes (Constantino et al., 2011).

## Author Contributions

MT conceived of the idea and wrote the initial draft of the article. Both authors worked on the revision and refinement of the paper and approved the final version.

### Conflict of Interest Statement

The authors declare that the research was conducted in the absence of any commercial or financial relationships that could be construed as a potential conflict of interest.
